# Dentistry in Its Relation to Medicine

**Published:** 1900-06

**Authors:** George S. Allan

**Affiliations:** New York


### DENTISTRY IN ITS RELATION TO MEDICINE.[1]

BY DR. GEORGE S. ALLAN, NEW YORK.

Mr. President and Gentlemen,—There are two topics, near to my heart and closely related, that I wished to hear mentioned: First, the enactment of laws compelling dentists to graduate as M.D.'s; second, the purification of our literature from trade influences. I regret that they have received so little attention.

A word in reference to the toast to which President Eliot so ably and thoughtfully responded. My respect for President Eliot and appreciation of the good work he has done in our cause is surpassed by no one here present. So, though you may, and quite likely will, question my judgment, you may not doubt my sincerity. But the wording of the toast strikes me unpleasantly,—"The Newness of Dentistry." Dentistry is not a new calling. History, ancient and modern, is full of references to it, quite as much so as to medicine in general.

Dentistry as a science, an art, or a profession has certainly rapidly advanced in late years. So has medicine. So has every specialty in medicine. All have shown a quickened life, a rapid advancement, and in about equal degrees. Dentistry is as old as the time when man had a body, and that body was subject to troubles, diseases, and torments. When Medicine(?) took the helm and said that she would attempt to alleviate these pains and sufferings, she did not say that she was going to take charge of one part of the body and not another part; that one part could be neglected and another part have close attention paid to it. The body and all that pertains to it, the mouth and the organs within it, are a most essential part of this connected whole. To be sure, dentistry occupies very peculiar relations to medicine in that its life and growth have not been connected with the parent profession. Dentistry is just as much a part of medicine as any other specialty; therefore I do not like the title, "Dentistry is New." Dentistry is simply seeking its rights. It wants to go where it belongs as an integral part of the medical profession, and never will it be properly represented until it is recognized as such the

[1] Read before the union meeting of The New York Institute of Stomatology and the American Academy of Dental Science, Boston, March 21, 1900.

world over. I know it is difficult to amalgamate it. It commenced its course outside of the medical profession, and it is now seeking its proper position. Medical schools did not incorporate it as a part of their curriculum. They did not teach the dental art, the dental science; they did not lay stress upon the dental organs, the organs of the mouth and all that relates to them. It is a most absurd thought that it can stand alone. To proclaim this idea is to state its impossibility. Dentists must have a thorough knowledge of medicine, and just as thorough a knowledge of medicine as general practitioners or those devoted to any other specialty. So when I hear this sort of talk, that we are a profession of ourselves, it angers me; it is not so.

I would like very much to have heard something said to-night on how to bring about that amalgamation of dental instruction with the instruction that is given to those who intend to practise medicine as a whole. I know a great many of my brethren who insist upon a separate dental college. I hold that this is radically wrong. The dental practitioner must start in to take his medical course and branch off when the proper time comes to take his specialty. I do not know how it is to be done, but it seems to me the proper way to go to work is to insist upon the medical colleges in their course of instruction giving special courses; in other words, the man who is to practise any specialty in medicine should be obliged to take the medical course as a whole for one, two, or three years, and then to have special instruction in whatever branches he chooses to take up. I do not know of any other way in which it can be done, but that the day will come when that, or some other equally practical course, will be adopted, I have no doubt.

We have had a most enjoyable and instructive evening. When you gentlemen come to New York I have not the slightest doubt but that all the members of the Institute will extend you a most cordial reception, and if we do not give you as good a time as you have given us, it will not be the fault of our intentions, it will be because we cannot do it, that New York does not afford the opportunities.

